# Interobserver and intraobserver agreement of ligamentous injuries on conventional MRI after simple elbow dislocation

**DOI:** 10.1186/s12891-017-1451-2

**Published:** 2017-02-20

**Authors:** Marc Schnetzke, Svenja Schüler, Johannes Hoffend, Rainer Simon, Holger Keil, Felix Porschke, Stefan Studier-Fischer, Paul-Alfred Grützner, Thorsten Guehring

**Affiliations:** 1 Clinic for Trauma and Orthopaedic Surgery, BG Trauma Center Ludwigshafen at Heidelberg University Hospital, Ludwig-Guttmann-Strasse 13, Ludwigshafen on the Rhine, 67071 Germany; 2 Institute of Medical Biometry and Informatics, University of Heidelberg, Heidelberg, Germany; 3 Department for Radiology, Klinikum der Stadt Ludwigshafen gGmbH, Ludwigshafen on the Rhine, Germany

**Keywords:** Elbow dislocation, Simple elbow dislocation, MRI, Interobserver, Intraobserver, Coronal oblique view, Medial collateral ligaments, Lateral collateral ligaments, Lateral ulnar collateral ligament

## Abstract

**Background:**

The primary objective of this study was to assess the interobserver and intraobserver agreement on ligamentous injuries on conventional magnetic resonance imaging (MRI) in acute simple elbow dislocation. The secondary objectives were to determine the interobserver agreement on the assessment of joint congruity, joint effusion, loose bodies and chondral lesions on conventional MRI.

**Methods:**

Conventional MRIs (1.5 Tesla, elbow specific surface coil) of 30 patients (40.7 years; range 14–72) with simple elbow dislocations were evaluated by four blinded examiners. An analysis of the interobserver agreement of all raters and for several subgroups (radiologists, orthopaedics, experienced, non-experienced) was performed. The examiners assessed the integrity (intact, partial tear, complete tear) of the lateral collateral ligament (LCL), medial collateral ligament (MCL), extensor and flexor tendons, as well as the presence of joint congruity, joint effusion, loose bodies and chondral lesions. Agreement strength, correlation and proportion of exact agreement were determined for interobserver agreement, and intraobserver agreement analyses.

**Results:**

Interobserver agreement of all examiners was fair to moderate for collateral ligaments (LCL: 0.441, MCL: 0.275). Exact agreement of all raters was found in 33.3% for the LCL and in 26.7% for the MCL. The both experienced examiners showed highest agreement strength for the LCL (0.619) and the radiologists showed highest agreement strength for the MCL (0.627), the proportion of exact agreement was 60.0% in both categories. A high proportion of exact agreement regarding joint congruity (90%), joint effusion (100%), loose bodies (96.7%) and chondral lesion (80%) was found among the radiologists. The evaluation of the intraobserver agreement revealed slight to substantial agreement (0.227 to 0.718) for the collateral ligaments.

**Conclusions:**

This study shows difficulties in the evaluation of ligaments by conventional MRI technique as demonstrated by a weak inter- and intraobserver agreement. This should be the basis to develop new MRI quality standards with special focus on coronal oblique reconstructions to improve the evaluation of ligament injuries after simple elbow dislocations.

## Background

Simple elbow dislocations are characterised by a severe soft tissue injury with a varying degree of instability [1–7]. The majority of simple elbow dislocations are stable after joint reduction and can be treated by exercises and functional use of the arm within 2 weeks of injury [3, 8, 9]. However, elbow dislocations can result in disabling sequelae, including recurrent instability, posttraumatic contractures and arthritis [10, 11]. Especially in unstable simple elbow dislocations, adequate treatment is still under discussion [6, 12, 13]. The treatment decision is mainly based on clinical and radiological findings [14]. Various imaging modalities can be used to determine the amount of the soft tissue injury and elbow instability, including ultrasound, plain radiographs, stress radiographs and magnetic resonance imaging (MRI) [12, 15–18]. However, a standardised diagnostic algorithm for acute simple elbow dislocations has not been established yet.

In chronic elbow instability the MRI has been proven to be a valuable diagnostic tool to detect full thickness tears of the collateral ligaments [19]. In acute simple elbow dislocation evidence for the reliability of the MRI is lacking. There are several concerns regarding its interpretation in ligamentous elbow injuries. Partial tears of the medial collateral ligaments (MCL) are detected with a low sensitivity [19, 20]. Biomechanically relevant structures like the lateral ulnar collateral ligament (LUCL) are not visualised reliably on MRI [21–24]. Furthermore, in acute simple elbow dislocation, most patients are not able to fulfil complete extension of the elbow, which makes interpretation much more difficult because the collateral ligaments are not tensioned [12, 25]. Therefore, the interpretation of an MRI after elbow dislocation might be difficult. The primary hypothesis of this study was that the interobserver and intraobserver agreement on ligamentous injuries on conventional MRI in acute simple elbow dislocation is weak. Secondary it was hypothesized that a high proportion of exact agreement on the assessment of joint congruity, joint effusion, loose bodies and chondral lesions on conventional MRI can be achieved.

## Methods

In this diagnostic study thirty consecutive patients with the diagnosis of a simple elbow dislocation who underwent an MRI within 4 weeks after injury were included between 2010 and 2015. The definition of simple elbow dislocation included patients with ligamentous injuries of the lateral (LCL) and/or medial collateral ligaments (MCL). Avulsion fractures of the coronoid process type I according to Regan & Morrey were also included in this study. This definition is in agreement with others [9]. Patients with avulsion fractures of the collateral ligaments, associated articular fractures of the radial head, the olecranon or coronoid fractures type II and III according to Regan & Morrey were excluded. Patients with previous injuries of the injured elbow were also excluded.

### Evaluation of MRI

The MRIs were examined by two blinded radiologists and two blinded orthopaedic surgeons. The evaluators experience and the average number of weekly-assessed MR examinations of the elbow and other major joints were assessed before the study (Table 1). The principal investigator selected the cases and knew the patients’ identities. The physicians evaluating the images were blinded to the diagnosis of patients.

**Table 1:** Length of experience and frequency of MRI examination evaluation of the elbow and other joints

Observer	Experience [y]	Elbow MRIs/week	Joint MRIs/week
Radiologist 1	29	10–15	100
Radiologist 2	25	1–5	10
Orthopaedist 1	15	15–20	60
Orthopaedist 2	5	1–5	10

The evaluators had access to the complete examinations, with the full sets of images. In addition, a short medical history and the time between injury and MRI were given to the evaluators. Subsequently, all evaluators were requested to fill out a questionnaire with eight items (Table 2).

**Table 2:** Questionnaire for the assessment of the MRI

Object	Detailed	Possible answers (No. of answers)
Soft tissue	Lateral collateral ligament (LCL)	Intact–partial tear–complete tear (3)
	Medial collateral ligament (MCL)	
	Common extensor tendon	
	Common flexor tendon	
Others	Joint congruity	Yes–no (2)
	Joint effusion	
	Loose bodies	
	Chondral lesion	

All MRIs were performed using 1.5 Tesla scanner with dedicated elbow specific surface coils. In each case, coronal, axial and sagittal images were available in non-fat-saturated T1-weighted and proton density–weighted sequences as well as fat-saturated T2/proton density–weighted or short tau inversion recovery (STIR) sequences. Special MRI reconstructions such as coronal oblique images have not been done. The evaluation of the MRIs was done on a medical viewing monitor with an adjustable brightness and contrast control.

### Statistical analysis

For characterising the study population descriptive statistics (mean, range, absolute and relative frequencies) are reported. An analysis of the interobserver agreement of all raters and for several subgroups (radiologists, orthopaedics, experienced, non-experienced) was performed. Both raters with routine evaluation of 10 or more MRIs/week were defined as experienced (radiologist 1 and orthopaedist 1) and the others that evaluate up to 5 MRI per week have been defined as non-experienced examiners (radiologist 2 and orthopaedist 2).

One option for evaluating the interobserver agreement is to estimate the proportion of exact agreement of all observers. This, however, does not take into account that observers will sometimes agree or disagree simply by chance. The kappa statistics are the most commonly used measure of agreement that take the expected agreement into account [26].

*Cohen’s kappa* was introduced to assess the interobserver agreement of two observers for categorical items. W*eighted kappa*, a generalised version of Cohen’s kappa, was designed to recognise that some disagreements between the two raters are more severe than others and thus weights disagreements differently (e.g. linear or squared). Therefore, it is especially useful for data measured on ordinal scales. In case of more than two raters assigning categorical outcomes, a further modification, *Fleiss’ kappa* is used to assess the agreement.

To evaluate the interobserver agreement of all raters or in the subgroups we therefore calculated Fleiss kappa or weighted kappa (squared weights) values respectively. Spearman’s rank correlation coefficients were calculated to identify relations between the different ratings in the subgroups. In addition the proportion of exact agreement was estimated. The interobserver agreement for the LCL and MCL for all observers was also analysed for different joint elbow positions in MRI (flexed vs. extended).

The intraobserver agreement assessed by w*eighted kappa* has been evaluated for the Orthopaedists comparing the results of the same examiner’s evaluations at 2 different times. Our target was to perform retests 2 months after baseline assessment. According to literature the time interval should be at least 2 weeks to prevent recall bias [27].

Agreement strength was inferred from kappa index values in accordance with the recommendations of Landis and Koch [28]. Briefly, a kappa value <0 was interpreted as poor agreement; a value in the range of 0.01 to 0.20, slight agreement; a value in the range of 0.21 to 0.40, fair agreement; a value in the range of 0.41 to 0.60, moderate agreement; a value in the range of 0.61 to 0.80, substantial agreement; and a value in the range of 0.81 to 1.00, nearly perfect agreement. Statistical analysis was performed using R version 3.1.3.

## Results

The sample consisted of 11 women (36.7%) and 19 men (63.3%) with an average age of 40.7 years (range 14 to 72). The left side was affected in 17 patients (56.7%) and the right side was affected in 13 patients (43.3%). The MRIs were performed 6.3 days (0 to 25) after injury. Twelve MRIs have been performed in our institution and 18 MRI have been performed externally. In 16 patients (53%) the elbow was in extended position and in 14 patients (47%) in flexed position during MRI examination.

The interobserver agreement and Spearman’s rank correlation coefficients for all subgroups are reported in Table 3. Interobserver agreement of all examiners was fair to moderate for collateral ligaments (LCL: 0.441, MCL: 0.275). Exact agreement of all raters was found in 33.3% for the LCL and in 26.7% for the MCL. The both experienced examiners showed highest agreement strength for the LCL (0.619) and the radiologists showed highest agreement strength for the MCL (0.627), the proportion of exact agreement was 60.0% in both categories.

**Table. 3:** Results of interobserver (κ_F_ = Fleiss Kappa, κ_w_ = weighted Kappa) and Spearman correlation (corr) (*κ could not be estimated due to spare data), bold values indicate substantial agreement

*N* = 30	All raters (*n* = 4)	Radiologists (*n* = 2)		Orthopaedists (*n* = 2)		Experienced (*n* = 2)		Non-Experienced (*n* = 2)	
	κ_F_	κ_w_	corr	κ_w_	corr	κ_w_	corr	κ_w_	corr
Ligaments									
LCL	0.441	0.570	0.625	0.590	0.636	**0.619**	0.652	0.467	0.554
MCL	0.275	**0.627**	0.631	0.498	0.567	0.547	0.575	0.185	0.183
Ext. tendon	0.049	0.093	0.133	0.309	0.215	0.363	0.323	0.089	0.212
Flex. tendon	0.143	*	0.043	0.175	0.090	0.351	0.359	0.121	0.119

**Fig. 1:**
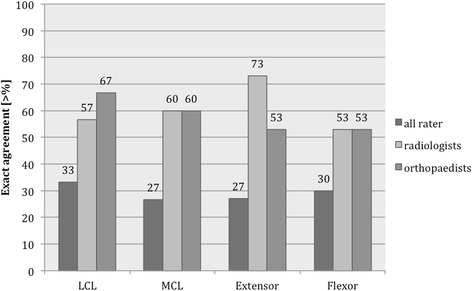
Proportion of exact agreement [%] for ligamentous structures

**Fig. 2:**
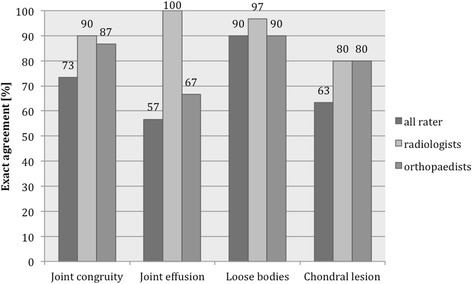
Proportion of exact agreement [%] for joint congruity, joint effusion, loose bodies and chondral lesion

An extended elbow position during MRI examination did not improve the interobserver agreement regarding LCL (extended: 0.359, flexed: 0.534) and MCL (extended: 0.284, flexed: 0.188). For binary variables (joint congruity, joint effusion, loose bodies and chondral lesion) interobserver agreement and Spearman’s rank correlation coefficients could not be estimated in most cases due to spare data. A high proportion of exact agreement regarding joint congruity (90%), joint effusion (100%), loose bodies (96.7%) and chondral lesion (80%) was found among the radiologists (Figs. 1 and 2).

**Fig. 3:**
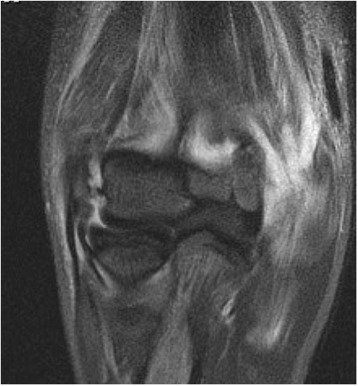
Coronal view in T2-weighted MRI after simple elbow dislocation: The LCL was rated to be partially torn (2×) and completely torn (2×)

**Fig. 4:**
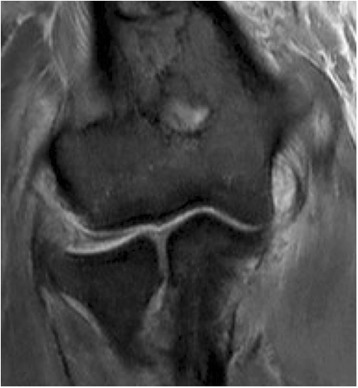
Coronal view in T2-weighted MRI after simple elbow dislocation: The MCL was rated to be intact (1×), partially torn (1×) and completely torn (2×)

The evaluation of the intraobserver agreement revealed slight to substantial agreement (0.227 to 0.718) for the collateral ligaments (Table 4). Patient examples with discrepancies regarding assessment of the LCL and MCL are shown in Figs. 3 and 4.

**Table 4:** Results of intraobserver agreement (κ_w_ = weighted Kappa, % = exact agreement), bold values indicate substantial agreement

*N* = 30	Orthopaedist 1		Orthopaedist 2	
	κ_w_	%	κ_w_	%
Soft tissue				
LCL	0.519	50	**0.718**	73
MCL	0.440	50	0.227	46
Ext. tendon	0.519	70	0.485	70
Flex. tendon	0.586	70	0.447	60

## Discussion

The most important finding of the current study was that interobserver agreement of all examiners was only fair to moderate for collateral ligaments (LCL: 0.441, MCL: 0.275) on conventional MRI after acute simple elbow dislocation. Subgroup analysis revealed that the experienced raters showed highest agreement strength for the LCL (0.619) and the radiologists showed highest agreement strength for the MCL (0.627). However, the proportion of exact agreement was still only 60.0% in both categories. Intraobserver agreement also showed only slight to moderate agreement for the collateral ligaments (0.188 to 0.586) with the exception of the rating of the MCL of orthopaedist No. 2 (0.718). The primary hypothesis was proven partially as most agreement analyses revealed only slight to moderate agreement on collateral ligament injuries. These results demonstrate that even for experienced radiologists and orthopaedists, the interpretation of an MRI after acute elbow dislocation might be difficult, particularly for detecting ligamentous injuries.

The number of categories and the grading system of injury will affect the results of any agreement analysis. In the current study, the grading system was adopted from a previous MRI study on the agreement of collateral ligament injuries [19]. However, no standardized classification system for the assessment of collateral ligament injuries on MRI exists. The lack of a classification system might be one reason for the low interobserver and intraobserver agreement found in this study.

There are several studies available regarding the diagnostic value of MRI for detecting tears of the ligamentous structures of the elbow. Previous studies assessing MRI of the elbow are limited by small numbers of patients and small numbers of patients who went on to surgery [19, 20, 29–32]. Furthermore, all previous studies regarding the usefulness of MRI in elbow injuries have been done in cadaveric models or chronic elbow instability.

Carrino et al. found only moderate interobserver agreement for detecting LUCL tears in a cadaveric model and concluded, that radial collateral ligament complex tears can be challenging to see on conventional MRI because of the oblique course of the ligament and the relatively small size of the ligament compared with the ulnar collateral ligament [29].

Carrino et al. conducted another cadaveric study with artificial tears of the MCL and found good agreement (0.78) [30]. Timmerman et al. found MRI to be 100% sensitive for full-thickness tears of the MCL but only 14% sensitive for partial-thickness tears in patients with chronic medial elbow pain; the specificity was 100% [19].

In the current study the LCL and the LUCL were not analysed separately, which would have further decreased the inter- and intraobserver agreement. The assessment of the LUCL can be difficult, and even the intact LUCL is rarely seen as a distinct low-signal band [21].

The assessment of extensor and flexor tendon injuries showed slight interobserver agreement of all raters (Ext. tendon: 0.049, Flex. tendon: 0.143). According to these results, distinguishing between injuries of the muscle tendons and the collateral ligaments seems to be even more difficult. However, determination of the muscle tendon injuries is an important aspect after simple elbow dislocation, because the muscle tendons are significant stabilisers of the elbow [33].

The discrepancies in the MRI findings regarding the ligamentous structures in our study lead to the question as to whether a conventional MRI is a valuable diagnostic tool in acute simple elbow dislocations. To improve the quality of MRI in acute elbow injury additional planes such as the posterior oblique coronal plane might be useful. In 1997, Cotten et al. investigated a cadaveric study to determine the best plane and position of the elbow for optimal visualization of normal and abnormal collateral ligaments with conventional MRI [34]. The authors concluded that the posterior oblique coronal plane with the elbows extended or the coronal plane aligned with the humeral shaft with the elbows slightly flexed allows accurate assessment of the collateral ligaments. Hill et al. also showed in a cadaveric study, that the medial collateral ligaments of the elbow were best seen on slight posteriorly oblique coronal plane as well [32]. In the current study, posterior oblique coronal views were not used. It should be the focus of further studies investigating the diagnostic accuracy of MRI in acute elbow dislocation using additional coronal oblique reconstructions. Improving the reliability of the assessment of important primary stabilizes such as the MCL and the LCL on the MRI could help the surgeon to decide on whether to treat the patients by functional rehabilitation alone or by primary surgical stabilization after simple elbow dislocation.

Until now, a diagnostic algorithm for simple elbow dislocation has not been established [12]. Sanchez-Sotelo et al. in 2005 reported that, if the clinical diagnosis is in doubt, a fluoroscopic examination should be performed and occasionally an examination under anesthesia, but other imaging studies such as MRI are usually not needed in simple elbow dislocations [35]. In contrast, Hackl et al. and our study group could show, that it is important to detect possible warning signs of instability on MRI after acute simple elbow dislocation [9, 36]. Our study group could show that the presence of a warning sign of instability (drop sign or joint incongruence) is associated with more complications (Odds ratio 15.9) and higher revision rates (Odds ratio 10.3) after non-operative treatment of simple elbow dislocations [9]. As hypothesized, a high proportion of exact agreement of all evaluators for joint congruity (73.3%) and for loose bodies (90%) has been found. The radiologist achieved exact agreement for joint congruity in 90%, for joint effusion in 100% for loose bodies in 97% and for chondral lesion in 80% of cases. This in agreement Hackl et al., who recently reported that tears of the LUCL with resulting posterolateral rotatory instability can be reliably diagnosed on MRI by assessing the joint congruity [36]. These findings support the notion that MRI in combination with clinical finding and stress radiography plays an important role in acute simple elbow dislocation.

This study has several limitations. MRI findings were not compared with intraoperative findings. Therefore, further prospective studies are necessary to evaluate the value of the MRI in simple elbow dislocation and to compare the assessment of the evaluators with intraoperative findings. Another limitation of this study is that intraobserver agreement was only available for the orthopaedic surgeons. In the current study conventional MRIs without coronal oblique reconstructions were used in all patients, which might have been improved the quality of MRI, and the interobserver and intraobserver agreement of the assessment of the ligamentous structures. Since this study was conducted in one single department, and the assessment was done by only four examiners, the findings may not necessarily be generalizable.

## Conclusions

This study shows difficulties in the evaluation of ligaments by conventional MRI technique as demonstrated by a weak inter- and intraobserver agreement. This should be the basis to develop new MRI quality standards with special focus on coronal oblique reconstructions to improve the evaluation of ligament injuries after simple elbow dislocations.

## Abbreviations

LCL: Lateral collateral ligaments
LUCL: Lateral ulnar collateral ligament
MCL: Medial collateral ligaments
MRI: Magnetic resonance imaging
STIR: Short tau inversion recovery
